# Comparison of Microleakage and Penetration Depth between Hydrophilic and Hydrophobic Sealants in Primary Second Molar

**DOI:** 10.5005/jp-journals-10005-1380

**Published:** 2016-12-05

**Authors:** Pritesh N Gawali, Vishwas B Chaugule, Amey M Panse

**Affiliations:** 1 Postgraduate Student, Department of Pedodontics and Preventive Dentistry Sinhgad Dental College and Hospital, Pune, Maharashtra, India; 2 Professor and Head, Department of Pedodontics and Preventive Dentistry Sinhgad Dental College and Hospital, Pune, Maharashtra, India; 3 Senior Lecturer, Department of Pedodontics and Preventive Dentistry Sinhgad Dental College and Hospital, Pune, Maharashtra, India

**Keywords:** Fissure sealant, Microleakage, Penetration depth.

## Abstract

**Introduction:**

Optimal pit and fissure sealing is determined by surface preparation techniques and choice of materials. The performance of pit and fissure sealant materials has been intensively investigated, yet no single product is reported as an ideal sealant. In children, moisture control during cavity preparation is always a big challenge, and hence, hydrophilic sealants have been developed.

**Aim:**

To compare the microleakage and penetration depth of hydrophilic and hydrophobic sealants using acid-etching on dry and moist surfaces.

**Materials and methods:**

Recently, extracted 28 2nd primary molars are assigned to two groups (hydrophobic group I; hydrophilic group II) depending on the surface condition (dry group: A1 and B1; moist group: A2 and B2) of 7 teeth in each group. Samples from group A1 and B1 are cleaned and dried with a 3-way syringe and etched with etching gel, and sealant is applied to the fissures and cured with visible light. Sample from A2 and B2 are immersed in 0.1 mL of fresh whole human saliva for 20 seconds and dried using a pellet cotton, and the same procedure is carried out. All samples are subjected to 1000 thermal cycles and sectioned to compare the depth of penetration and microleakage. Sections will be examined under light microscope and analyzed using an image analysis software (SigmaScan).

**Results:**

The least microleakage was seen with hydrophilic sealant under moist surface condition, and the depth of penetration of hydrophobic sealant was found to be better than that of hydrophilic sealant in both dry and moist surface conditions.

**Conclusion:**

Hydrophilic pit and fissure sealants showed higher tolerance to saliva contamination with less microleakage, but in terms of penetration ability hydrophobic sealants were found to be superior.

**How to cite this article:**

Gawali PN, Chaugule VB, Panse AM. Comparison of Microleakage and Penetration Depth between Hydrophilic and Hydrophobic Sealants in Primary Second Molar. Int J Clin Pediatr Dent 2016;9(4):291-295.

## INTRODUCTION

“Intellectuals solve problems; geniuses prevent them!”

Caries occur five times more frequently in occlusal fissures than on smooth surfaces. Inaccessible morphology of these areas prevents them from being cleaned properly, thereby increasing the potential for plaque accumulation. Occlusal caries account for 56 to 70% of the lesions in children aged between 5 and 17 years.^1^ Fissure sealing is an established and effective approach for caries management on occlusal surfaces.^2^ It is known that sealants form a mechanical barrier for microorganisms and plaque with obstruction of retentive pit and fissures.^3,4^ Application of fissure sealant have been shown to be an effective method for preventing caries and halting its progress.^3-6^ The dental battle against decay in pits and fissures has a long and creative past, which dates back when they were sealed with zinc phosphate cement, mechanical fissure eradication, prophylactic odontotomy, and chemical treatment with silver nitrate. Creativity in this effort against fissure caries continues, with new materials and technologies been tested each year. Dental sealants are effective in preventing dental caries in the occlusal and other pitted and fissured surfaces of the teeth.^7^ The important properties of an ideal sealing material include ability to seal and penetrate into the depth of the pits and fissure as well as retention and resistance to abrasion and wear. Lack of sealing or insufficient penetration of the material in the deep fissures allows the occurrence of marginal leakage through the tooth-material interface, which can promote caries lesion progression underneath the restoration leading to treatment failure.^8^ As moisture control in children is a big challenge, and which is the key factor for success of the preventive treatment regime, hydrophilic sealants are now introduced in the market. This article aims to study the behavior pattern of hydrophilic sealants in comparison to the conventional hydrophobic sealant in primary 2nd molars under dry and moist surface conditions.

**Fig. 1:**
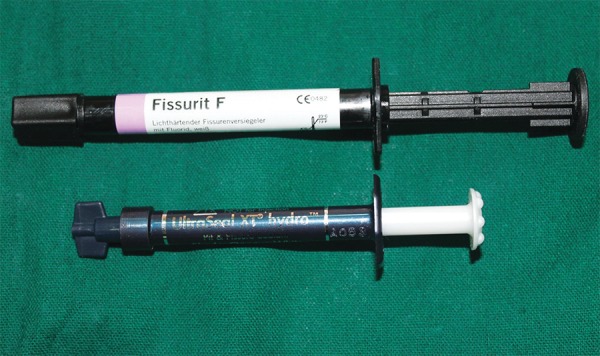
Test materials

**Fig. 2:**
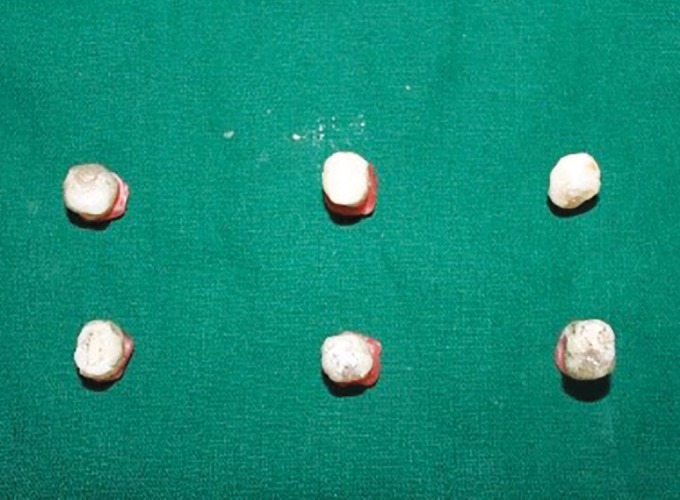
Application of sealant

## MATERIALS AND METHODS

Two pit and fissure sealants were used in this study. The following is the list of materials (Fig. 1) and armamentarium used in this study.

### Test Materials

- *Hydrophilic sealant:* UltraSeal XT hydro S3529 (manufacturer: UltraDent, Germany).
- *Hydrophobic sealant:* Fissurite F (manufacturer: Voco GmbH, Germany).

### Equipments

- Dental explorer
- Small round bur
- Applicator tips
- Stereomicroscope
- LED curing system Model 700, serial 19365.

Twenty-eight freshly extracted noncarious human primary mandibular 2nd molar teeth were selected for this study. All the collected teeth were obtained from children aged 9 to 11 years whose parents were fully informed about the study and from whom a written consent was obtained. After extraction, the teeth were stored in thymol 2% for 24 hours. Enameloplasty was performed using a cone-shaped bur, as described by Duangthip D et al.^9^ Just prior to sealing, the teeth were cleaned with a brush in a low-speed micromotor handpiece without pumice, and a dental explorer was used to clean debris from the pits and fissures. Occlusal surface of the teeth were itched with 37% phosphoric acid for 30 seconds and then rinsed with water and dried thoroughly. The teeth were later randomly divided into four groups of seven teeth in each (Table 1).

**Table 1:** **Table 1:** Grouping of teeth based on application of sealants under dry and moist surface condition

*Sealant*		*Surface condition*	
Hydrophilic (group A)		Dry A1 (n = 7)	
		Moist A2 (n = 7) (saliva contaminated)	
Hydrophobic (group B)		Dry B1 (n = 7)	
		Moist B2 (n = 7) (saliva contaminated)	

### Application of Sealant

Procedure for the Samples in Group A1 and B1 under Dry Surface Condition

Hydrophilic sealant (UltraSeal XT hydro S3529) and hydrophobic sealant (Fissurite F) were applied on the occlusal surface of the teeth in group A1 and B1 as per the manufactures instructions followed by curing with halogen light (LED curing system Model 700, serial 19365) for 20 seconds (Fig. 2).

Procedure for the Samples in Group A2 and B2 under Moist Surface Condition

**Fig. 3:**
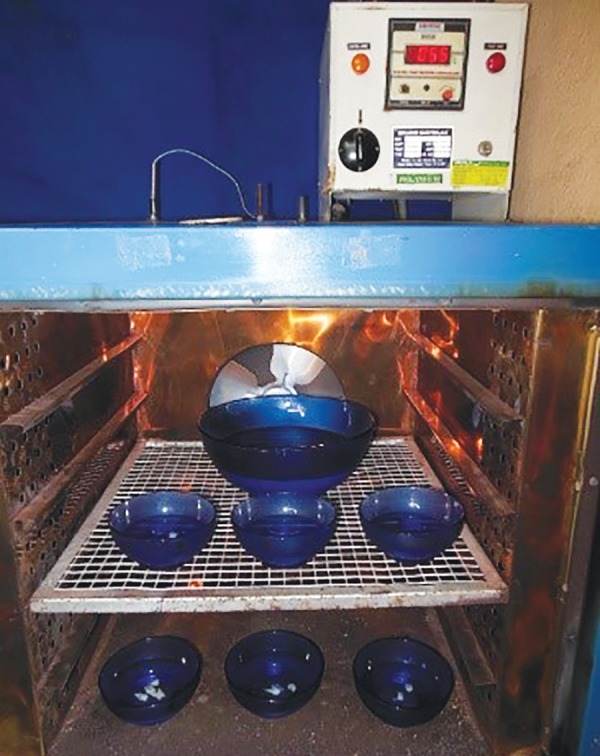
Thermocycling of samples

Occlusal surfaces of samples in this group were contaminated with 0.1 mL of fresh human saliva for 20 seconds and then dried using a cotton pellet for 5 seconds. Then both the sealants were applied and light cured for 20 seconds. In all groups, the sealant application was limited to the borders of the fissure, and a waiting period of 10 seconds was employed prior to light curing. After sealing, the teeth were kept in distilled water at 37°C for 24 hours. Subsequently, all the samples were submitted to a thermocycling regimen of 1000 cycles between 5 and 55°C water baths with a dwell time of 1 minute at each temperature (Fig. 3). The teeth were later embedded in resin to cover the apices, and all of the surfaces were covered with nail varnish except for 2 mm around the fissure margins.

### Dye Penetration and Sectioning

The samples were immersed in methylene blue 2% for 24 hours. Following dye exposure, teeth were washed and rinsed with distilled water, dried and sectioned with a water-cooled diamond disk in a mesiodistal plane through the sealant.

### Microscope Analysis

After sectioning, each section was then examined using a stereomicroscope at 10x magnification, and images were transferred to a personal computer to be analyzed for microleakage and penetration. The images showing microleakage and penetration depths were then analyzed using image analysis software [SigmaScan, Statistical Package for the Social Sciences (SPSS); Jandel Scientific, San Rafael, CA, USA].

The degree of microleakage was blindly scored by two independent examiners, using a grade scale;

Score 0 = No dye penetration

Score 1 = Dye penetration restricted to the outer half of the sealant

Score 2 = Dye penetration restricted to the inner half of the sealant

Score 3 = Dye penetration to the underlying fissure.

Nonparametric tests Kruskal-Wallis and Mann-Whitney tests were applied to the values obtained to compare the relationship among the different groups.

**Fig. 4:**
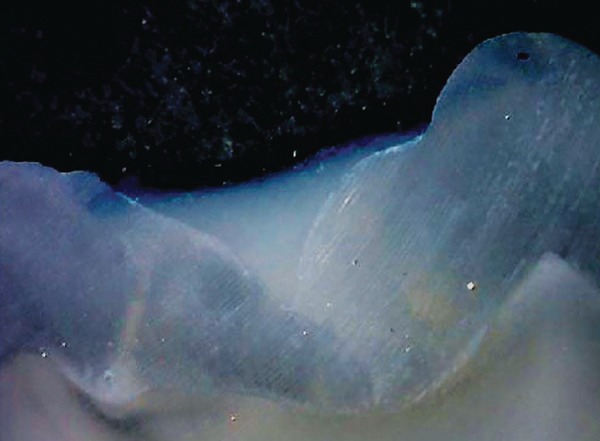
Score 0

**Fig. 5:**
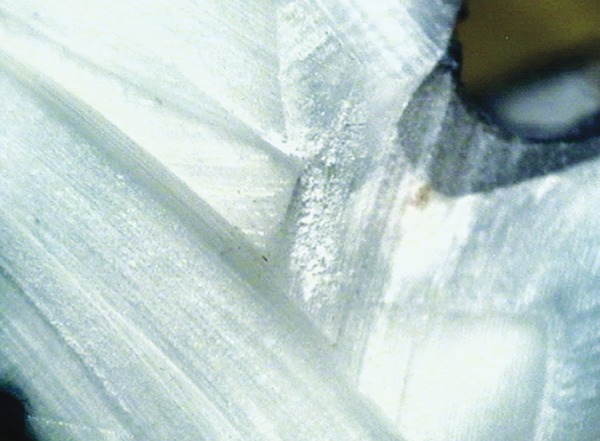
Score 1

**Fig. 6:**
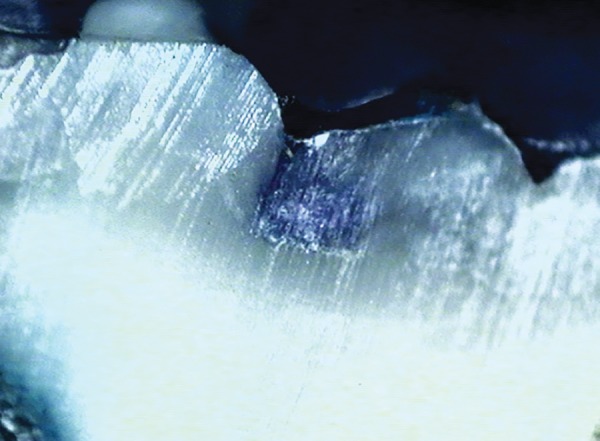
Score 2

## RESULTS

A total of 28 sections were examined for microleakage and penetration depth.

**Fig. 7:**
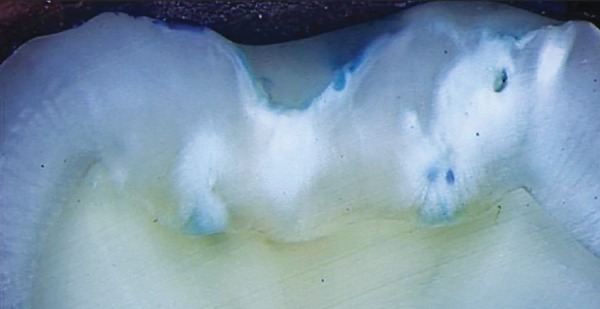
Score 3

Table 1 shows comparison of values obtained for microleakage. Images for the different scores of micro-leakage is shown in Figures 4 to 7.

No significant difference was seen in the microleakage between following groups (p > 0.05).

A1 *vs* A2

B1 *vs* B2

A1 *vs* B1

However significant difference was seen between the group A2 *vs* B2 (p = 0.024).

Both sealants used in this study showed some degree of microleakage in dry and moist conditions.

The least microleakage was seen with hydrophilic sealant under moist surface condition.

**Graph 1:**
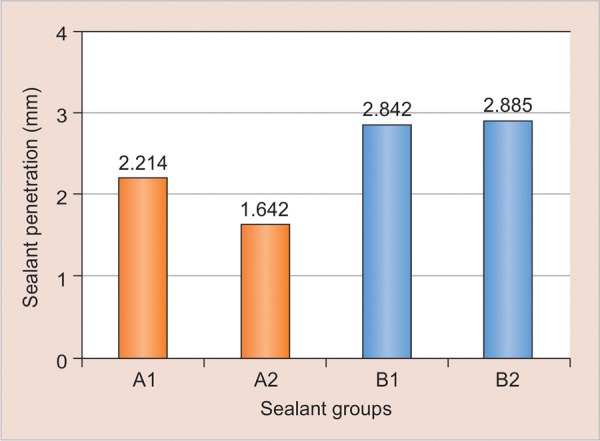
Depth of penetration of both sealants under dry and moist surface condition

Graph 1 shows the levels of depth penetration of two sealants under dry and moist conditions.

Significant difference was seen in the penetration depth of all the four groups:

A1 *vs* A2 (p = 0.007)

B1 *vs* B2 (p = 1.000)

A1 *vs* B1 (p = 0.038)

A2 *vs* B2 (p = 0.007)

From the results, it can be concluded that the depth of penetration of hydrophobic sealant was found to be better than that of hydrophilic sealant in both dry and moist surface conditions.

## DISCUSSION

Pit and fissure sealants have been considered an outstanding adjunct to oral health care preventive strategies in the decrease of occlusal caries onset and or progression. Occlusal pits and fissures vary in shape, but are generally narrow and tortuous, and are considered to be an ideal site for retention of food remnants, providing an ideal habitat for bacteria. In children, primary 2nd molar is the most susceptible for pit and fissure caries. Therefore, to prevent initiation of caries in these fissures, the concept of pit and fissure sealants evolved. For retention, it is important to adequately isolate the teeth. Salivary contamination is the major cause for the loss of sealants in the 1st year, especially in children where is difficult to achieve isolation. The conventional hydrophobic sealants have shown increased microleakage and decreased bond strength in saliva-contaminated fissures, and hence hydro-philic sealants were introduced. The marginal sealing is important for sealant success because penetration of bacteria beneath the sealant might allow caries onset and/or progression.^10^ Study on pit and fissure sealants by Cueto and Buonocore^11^ reported 87% caries reduction and 71% complete retention of the sealant material on primary molars. Although some researchers showed no significant difference in microleakage when a bur enameloplasty or solely phosphoric acid-etching was used prior to pit and fissure sealant application,^12,13^ others reported the superiority of the former technique;^14,15^ hence, enamelo-plasty was performed in the present study. The results of this study revealed that hydrophilic sealant under moist surface condition exhibited the least microleakage, which was in contrast with the study done on permanent molars by Khogli et al,^16^ which concluded that microleakage was highest with hydrophilic sealants under moist surface conditions. Thus least microleakage under moist conditions indicates that the presence of moisture did not interfere in the better adaptability of sealant to the fissure. In an *in vitro* study on hydrophobic and hydrophilic sealants by Baagherian,^17^ it was suggested that from the micro-leakage aspect, the hydrophilic sealant may be used as an acceptable alternative to the hydrophobic sealant, which was in accordance with the current study but in primary molars. The penetration depth is also a very important parameter which may affect the sealant retention. Several factors, such as the morphology of the fissures^18^ and properties of the materials^18^ have been suggested to have an influence on the penetration ability of pit and fissure sealants. Addition of filler particles lowers sealants’ ability to penetrate into deep fissures and microporosities of etched enamel. Hence, in our study, penetration depth was superior for unfilled hydrophobic sealant (Fissurite F) under dry and moist surface conditions, showing better sealing ability for the same. One of the limitations of this study was the fact that our study was an *in vitro* evaluation, and moisture control was easy to achieve. But the fact is that even when stringent moisture control procedures are attempted during sealant application, contamination can occur. In addition, minute contamination also occurs in other areas where total isolation is not possible, such as buccal or lingual grooves. These contaminations are the likely cause of the sealant failure. Therefore, to keep the uniformity within the samples, all the samples in the study were acid-etched. In addition, *in vivo* investigations are necessary. In this context, parameters, such as long-term retention and shear bond strength of hydrophilic sealants must be considered.

## CONCLUSION

The present study showed higher tolerance of the newer hydrophilic pit and fissure sealant to saliva contamination with less microleakage, but in terms of penetration ability the newer sealant was found to be inferior to the conventional sealants. Clinical studies reporting on sealant success, when applied to primary molars, are rare. Further *in vivo* and *in vitro* studies with larger sample size need to be carried out to evaluate the material further. Nevertheless, despite its limitations, this study provides some data to support further interest into the use of hydrophilic fissure sealant in pediatric dentistry.
